# A Community-Based Usability Study of an AI-Enabled Oral Cancer Screening App Operated by Village Health Volunteers: Mixed Methods Study

**DOI:** 10.2196/83738

**Published:** 2026-03-09

**Authors:** Mansuang Wongsapai, Kornwipa Wudtijureepun, Thawatchai Suthachai, Decha Tamdee, Jitjiroj Ittichaicharoen, Patiwet Wuttisarnwattana, Siriwan Suebnukarn

**Affiliations:** 1 Ministry of Public Health of Thailand, Intercountry Centre for Oral Health, Chiang Mai, Thailand; 2 Faculty of Nursing, Chiang Mai University, Chiang Mai, Thailand; 3 Faculty of Dentistry, Chiang Mai University, Chiang Mai, Thailand; 4 Faculty of Engineering, Chiang Mai University, Chiang Mai, Thailand; 5 Faculty of Dentistry, Thammasat University, Piyachart Building, 9th Floor, Phaholyothin Road, Pathum Thani, 12120, Thailand, 66 29869213, 66 29869205

**Keywords:** mobile apps, cancer screenings, prevention, early detection of cancer, health inequity

## Abstract

**Background:**

Oral cancer is a major public health concern in low- and middle-income countries, where access to specialist care and early detection remains limited. Mobile health technologies supported by artificial intelligence (AI) offer a scalable approach to extend screening services into underserved communities. In Thailand, village health volunteers (VHVs) are key frontline workers who provide preventive services and bridge gaps between rural populations and specialist care.

**Objective:**

This study aimed to describe the technical development of RiskOCA (Risk Assessment for Oral Cancer using Artificial Intelligence), a smartphone-based, AI-assisted oral cancer screening platform, and evaluate its usability when deployed by VHVs in a rural Thai province.

**Methods:**

RiskOCA was developed using a 3-tier architecture comprising a patient-facing interface for risk factor profiling and guided imaging, an embedded deep learning engine (DeepLab v3+with ResNet-50 backbone) for lesion detection, classification, and segmentation, and a secure specialist portal for expert review of all cases. The AI model was trained on 2226 annotated intraoral images and validated for real-world use. Field testing was conducted in the Phu Kamyao district, Phayao province, where 1242 adults (≥40 y) were screened with assistance from VHVs. Usability was evaluated through a structured 25-item questionnaire completed by 250 VHVs, with responses rated on a 5-point Likert scale.

**Results:**

The AI model achieved a mean classification accuracy of 93.22% (SD 0.88%) across 3 diagnostic categories. Usability evaluation indicated high satisfaction across all domains, with an overall mean score of 4.17 out of 5. The highest ratings were for the app’s impact on older adult surveillance (mean 4.30), while all domains were rated “satisfied” or “very satisfied.”

**Conclusions:**

RiskOCA demonstrated strong technical performance and high user acceptance among VHVs, supporting its feasibility for community-based oral cancer screening. By integrating AI-assisted triage with expert review, the platform has the potential to reduce diagnostic delays, expand screening coverage, and serve as a scalable model for oral cancer prevention in resource-limited settings.

## Introduction

### Background

Oral cancer continues to be a major global health burden, particularly in low- and middle-income countries where access to early detection and specialist care remains limited. In Southeast Asia, oral health is often underprioritized in public health policy, despite its growing impact on population well-being. In 2020, the region reported more than 900 million cases of oral disease and nearly 100,000 deaths due to lip and oral cavity cancers—conditions that disproportionately affect rural populations [1]. In response, the World Health Assembly and the World Health Organization Southeast Asia Regional Committee have called for oral health to be integrated into universal health coverage and noncommunicable disease frameworks [2]. Nevertheless, geographic, financial, and infrastructural barriers continue to prevent timely access to screening and care in remote communities.

The early detection of oral squamous cell carcinoma (OSCC) and oral potentially malignant disorders (OPMD) is essential to reducing disease burden, improving survival outcomes, and preserving function. However, timely diagnosis is often hindered by low health literacy, insufficient clinical workforce capacity, and delayed referrals [3,4]. In rural Thailand and similar settings, the challenge lies not only in detection but also in delivering equitable access to specialist assessment and follow-up. One promising strategy to address this gap is task-shifting, wherein screening responsibilities are extended to trained community health workers—such as Thailand’s village health volunteers (VHVs)—who serve as trusted frontline actors in noncommunicable disease surveillance and public health promotion [5].

Thailand’s VHVs, recognized as a cornerstone of its universal health coverage success, play a pivotal role in linking underserved communities with formal health care systems [6]. Their responsibilities include conducting home visits, collecting health data, and facilitating screening initiatives across a range of conditions, including hypertension, diabetes, and cervical cancer. Leveraging this existing infrastructure for oral cancer screening could offer a scalable, sustainable approach to early detection—particularly if supported by digital health tools that enable accurate assessment and specialist consultation.

Mobile health (mHealth) technologies have emerged as effective solutions to expand health care access in resource-limited settings. Mobile apps can support self-screening, risk stratification, and community-based triage and have shown promise across various domains, including infectious disease surveillance and chronic care [7,8]. In the context of oral cancer, smartphone-based platforms can enable nonspecialists to identify suspicious lesions and trigger referral pathways earlier in the disease course. Yet, despite advances in design and accuracy, few tools have been evaluated for usability or real-world effectiveness in frontline settings, particularly among nonexpert users, such as VHVs [9].

Recent developments in artificial intelligence (AI)—especially deep learning—have demonstrated strong diagnostic potential in analyzing clinical images of oral lesions [10,11]. However, most AI models remain isolated in research settings, with limited translation to community health workflows. To have a public health impact, these technologies must be embedded within systems that are accessible, usable, and clinically meaningful for those operating outside hospital environments [12].

To address this challenge, we developed RiskOCA (Risk Assessment for Oral Cancer using Artificial Intelligence), a smartphone-based app designed to support AI-assisted screening and referral for oral cancer [13]. The platform integrates risk factor profiling, guided intraoral imaging, and lesion analysis via deep learning, with all submitted cases reviewed by oral cancer specialists. Crucially, RiskOCA was designed using a user-centered approach to enable VHVs—as well as other frontline workers—to participate in the early detection process, thereby bridging the gap between remote communities and oral cancer specialists.

### Objectives

This paper presents the technical development and usability evaluation of RiskOCA, with a focus on its system architecture, user interface design, and field-based testing among VHVs in a rural Thai province. By examining how VHVs interact with the app and assess its usability, the study provides insights into the potential of AI-integrated mHealth tools to strengthen community-based cancer screening and reduce diagnostic delays in resource-limited settings.

## Methods

### Ethical Considerations

This study comprised three main components: (1) system development, which included the architecture, AI model, platform technology stack, and data security protocols; (2) user interface and experience design, guided by a user-centered approach to ensure accessibility and cultural relevance; and (3) user evaluation, which involved field-based testing among community participants and VHVs in a rural Thai province. All procedures were conducted in accordance with ethical standards for research involving human participants.

The study was approved by the Regional Health Promotion Center 1, Department of Health, Ministry of Public Health, Thailand (approval No. 20/2565, dated April 29, 2022). Informed consent was obtained from all participants prior to participation using both written and verbal processes conducted in the local language (Thai). Written informed consent was documented using an Institutional Review Board–approved consent form. In addition, trained VHVs provided a standardized verbal explanation of the study purpose, procedures, potential risks and benefits, data handling practices, and the voluntary nature of participation.

The consent process explicitly informed participants that their clinical images and associated data would be used for AI-based risk assessment and may be included in anonymized datasets for research and model development. Participants were informed that no personally identifiable information (PII) would be used for AI training, that participation could be declined or withdrawn at any time without consequence, and that no financial compensation was provided.

Verbal consent was recorded by study staff using a consent checklist confirming that key information had been explained and understood. Participant understanding was assessed through interactive dialogue, during which participants were encouraged to ask questions and were asked to confirm their understanding of the core elements of the study, including data use and privacy protections, before enrollment proceeded.

To ensure confidentiality, all PII (eg, names, national ID numbers, and contact details) was removed or stored separately prior to analysis. The research team only had access to the fully anonymized data. Participants did not receive any compensation for their data being included in this study.

### System Development

#### Architecture Overview

RiskOCA is a smartphone-based digital platform designed to support oral cancer risk assessment and guided self-examination, particularly among adults in underserved and remote communities. The system architecture comprises four core components: (1) an individual risk profiling module, which collects personal health data and behavioral risk factors (eg, tobacco and alcohol use, betel nut chewing, denture wear); (2) a user-facing mobile web app, which guides individuals step by step in capturing high-quality intraoral images using smartphone cameras; (3) an embedded AI engine, a deep learning model that detects and segments potentially malignant lesions in submitted images; and (4) a specialist review portal, which enables trained oral medicine professionals to verify AI-generated findings and provide clinical recommendations.

Images flagged as high-risk by either the AI system or the reviewing specialist are referred for follow-up, which may include a clinical examination and histopathological confirmation. All user data are securely transmitted and stored in compliance with national ethical and privacy guidelines. By integrating automated triage, guided self-screening, expert validation, and follow-up pathways, RiskOCA offers a scalable framework to enhance the early detection and surveillance of oral cancer in low-resource settings.

#### Platform Technology Stack

RiskOCA was built on a 3-tier architecture, which separates the system into the presentation layer (frontend), app layer (business logic), and data layer (storage and management). This modular architecture facilitates scalability, maintainability, and efficient system updates.

At the presentation tier, the front-end interface was developed using the Ionic framework, enabling cross-platform deployment on both Android and iOS devices from a single codebase. The interface uses standard web technologies (HTML5, CSS3, and JavaScript) to ensure responsiveness and consistent user experience across device types.

The application tier incorporates an application programming interface layer implemented in PHP (version 7.4.5), which manages all business logic, handles interactions between the frontend and the backend, and processes communications with the AI engine and clinician portal.

The data tier is hosted on an Apache web server and uses a MySQL relational database for storing user records, clinical metadata, AI results, and clinician annotations. The database is configured with utf8_general_ci character encoding to support multilingual content, including Thai and English. Database management is performed via phpMyAdmin (version 5.0.2), which facilitates tasks, such as schema definition, data linking, and automated backups. Together, this stack—Ionic (user interface/user experience), PHP (logic or application programming interface), and MySQL (data storage)—provides a robust, scalable, and cost-effective foundation for RiskOCA’s deployment in real-world health systems.

#### AI Model Description and Performance

A central feature of RiskOCA is its integration of a deep learning model to enable automated, image-based screening for oral lesions. The model is based on the DeepLab v3+ architecture with a ResNet-50 backbone, a widely used configuration for semantic segmentation tasks. It was optimized for intraoral images captured in nonclinical environments, accommodating variability in lighting, positioning, and mucosal anatomy [14]. The AI system performs three key functions: (1) lesion detection—identifying the presence of any abnormal lesion in the image; (2) classification—categorizing lesions into one of 3 diagnostic classes: normal oral mucosa, OPMD, or OSCC; and (3) segmentation—generating a pixel-level mask that outlines the lesion boundaries to assist with visual interpretation and confirmatory review.

The dataset included 2591 images, each accompanied by expert-annotated segmentation masks. Images were acquired from multiple oral cavity regions using both clinical-grade single-lens reflex cameras and consumer mobile phone cameras, reflecting real-world variability in image acquisition. All images were resized to 342×512 pixels before being input to the model. To mitigate potential data leakage, substantially similar or near-duplicate intraoral photographs from the same participant were identified through manual review and consensus by 2 board-certified oral and maxillofacial pathologists and removed prior to model training and cross-validation. The model was finally trained and evaluated on 2226 labeled intraoral images (normal: n=667, OPMD: n=1310, OSCC: n=249).

A 5-fold stratified cross-validation was used to evaluate the robustness and generalizability of the DeepLab v3+model with a ResNet-50 backbone. In each fold, 20% of the data served as the held-out test set, while the remaining 80% were split into training (70%) and validation (10%). Model selection was based on validation loss, and the final performance for each fold was evaluated on the independent test set. To derive an image-level screening label from the segmentation output, a lightweight postprocessing rule was applied to summarize pixel-level predictions into one of the 3 diagnostic categories. Specifically, the predicted label was assigned according to the dominant predicted lesion class in the segmentation map, with the suppression of very small and isolated predicted regions to reduce spurious micro-segmentations. This strategy provides both an interpretable lesion outline and a single screening label suitable for community-based triage.

Across the 5 cross-validation folds, the model achieved an overall classification accuracy of 93.22 (SD 0.88%). Class-specific performance was stable and strong for normal and OPMD and clinically meaningful for OSCC. Normal classification achieved a precision of 90.82% (SD 2.41%), recall of 92.65% (SD 1.65%), and *F*_1_-score of 91.70% (SD 1.17%). OPMD classification achieved a precision of 94.96% (SD 0.67%), recall of 94.73% (SD 1.93%), and *F*_1_-score of 94.83% (SD 0.88%). For OSCC, the model achieved a precision of 90.78% (SD 2.16%), recall of 86.78% (SD 5.17%), and *F*_1_-score of 88.67% (SD 3.23%).

The pooled confusion matrix across all held-out test images from the 5 folds (n=2226) is shown in Table 1. Most images were correctly classified, with diagonal counts of 618 normal, 1241 OPMD, and 216 OSCC cases. The predominant OSCC error mode was misclassification as OPMD (24/249, 9.6%), whereas the misclassification of OSCC as normal occurred less frequently (9/249, 3.6%). In the context of community-based screening with specialist review, the misclassification of OSCC as OPMD would still flag abnormality and prompt follow-up, whereas OSCC misclassified as normal represents the more clinically concerning miss category.

**Table 1.** Pooled confusion matrix (all test images across 5 folds; counts).

Ground truth	Prediction		
Normal, n (%)	OPMD^a^, n (%)	OSCC^b^, n (%)
Normal	618 (92.7)	42 (6.3)	7 (1.0)
OPMD	54 (4.1)	1241 (94.7)	15 (1.2)
OSCC	9 (3.6)	24 (9.6)	216 (86.8)

a OPMD: oral potentially malignant disorders.

b OSCC: oral squamous cell carcinoma.

Segmentation performance was consistent across folds, with a pixel-wise micro mean intersection over union of 92.32% (SD 0.55%) and a micro mean *F*_1_-score of 96.01% (SD 0.30%). Per-class pixel intersection over union was the highest for normal (95.75%, SD 0.28%) and remained moderate for lesion classes (OPMD: 69.30%, SD 1.28%; OSCC: 65.15%, SD 4.34%). The lower and more variable intersection over union for OSCC indicates greater heterogeneity in lesion boundaries, which is expected given the diversity of tumor morphology and extent.

#### Security and Privacy

RiskOCA was designed with a security-first approach, emphasizing data confidentiality, integrity, and user consent. Prior to using the platform, participants must review and electronically consent to a clearly worded form detailing the study purpose, data handling policies, potential risks, and the voluntary nature of participation.

PII, including full name, national identification number, contact information, and demographic details, is collected during user registration to support clinical follow-up, referral coordination, and linkage with health care services. However, no PII is attached to image data at the point of image submission or during AI-based risk assessment. Instead, each participant is assigned a unique alphanumeric study ID, which is used to link clinical images and questionnaire data while decoupling them from direct personal identifiers.

To further protect privacy, all submitted images are stripped of embedded metadata, including GPS location, device identifiers, and timestamps, prior to storage and analysis. All data transmissions occur over HTTPS using TLS 1.3 encryption. PII and anonymized clinical data are stored in logically separated, encrypted MySQL databases, accessible only to authorized personnel under role-based access controls.

Clinical reviewers and AI developers are provided access only to anonymized cases and are not able to view names, national ID numbers, or contact information. All system access, AI outputs, and expert reviews are logged and auditable. RiskOCA complies with national data protection regulations and aligns with international best practices for health data governance. Participants may request the deletion of their data at any time via an embedded secure request form on the platform.

### User Interface and Experience Design

#### Patient Interface for Risk Assessment

The RiskOCA platform was designed using a user-centered design approach, with a strong emphasis on accessibility, cultural relevance, and ease of navigation for diverse user populations, particularly in rural and underserved areas. The patient-facing interface supports guided data entry, risk assessment, and health education through intuitive, mobile-optimized screens. The app is designed to be used independently by patients or with assistance from VHVs, ensuring inclusivity for users with varying levels of digital literacy.

**Figure 1.**
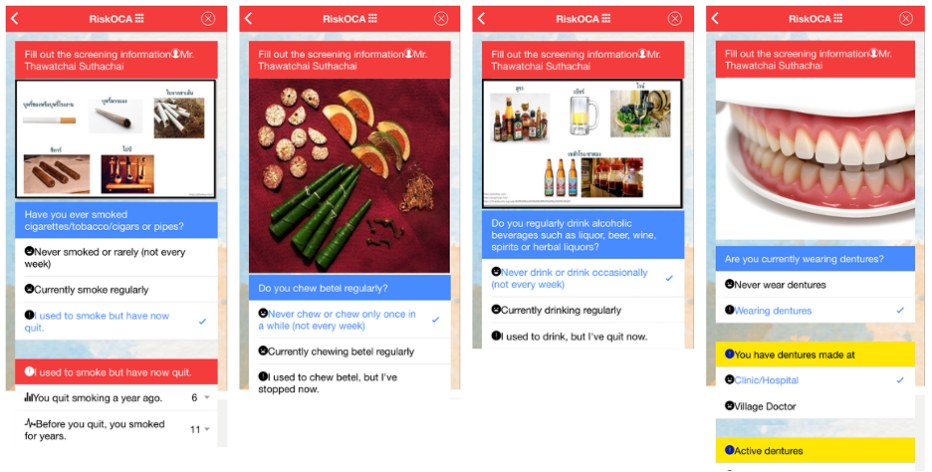
Oral cancer risk assessment user interface.

Upon launching the app, users are prompted to enter their full name and national ID number for clinical identification and follow-up purposes, which is essential for individuals undergoing screening for oral potentially malignant disorders or oral cancer. Real-time input validation is implemented to prevent incorrect or incomplete ID entry. Once validated, users proceed by selecting their province, district, and sub-district, and then enter additional demographic information, including phone number, gender, date of birth, and ethnicity. These entries are facilitated through standardized input menus, including dropdowns and scrollable selection wheels optimized for mobile devices (Figure 1).

Following demographic data entry, users are guided through a structured oral cancer risk assessment, consisting of four culturally and clinically relevant domains [15]:

1. Tobacco smoking: Users are asked if they have ever smoked cigarettes, cigars, pipes, or used tobacco leaves. Based on their response—“Never or only occasionally,” “Currently smoke regularly,” or “Used to smoke but quit”—the interface dynamically adapts to show follow-up questions. For current users, sliders allow the input of number of units smoked per day and duration of smoking (years). Former users are asked how many years ago they quit and for how long they smoked.
2. Betel nut chewing: Users indicate whether they have regularly chewed betel nut. If applicable, the app collects data on frequency per day and duration in years, again using slider-based inputs. Those who quit are asked about cessation timelines.
3. Alcohol consumption: The app asks whether the user currently drinks alcoholic beverages, such as spirits, beer, wine, or herbal liquors. For current drinkers, follow-up fields collect details about the type of alcohol, frequency (daily, weekly, and monthly), and duration. Former drinkers are asked to specify the duration of prior use and time since quitting.
4. Wearing dentures: Users report whether they currently wear dentures. Those who do are asked about the source of fabrication (clinic or hospital vs local practitioner), fit quality (well-fitting or loose), and duration of denture use in years.

Each question concludes with a visual confirmation indicator (✓) and a “Confirm” button, ensuring users understand and acknowledge their responses before proceeding.

After completing the risk assessment, the app displays personalized feedback based on the presence of any risk factors. If risk factors are identified, a warning message is shown: “You should receive an oral screening by a dentist, or dental health professional.” Users are prompted to enter their current address for follow-up and are provided with a link to self-examination instructions for oral cancer. If no risk factors are present, the message displayed is: “You are not in a risk group, but it is recommended to have an oral health check-up every 6 months.” The app then concludes with a thank-you message.

Throughout the interface, the design prioritizes simplicity and responsiveness, using mobile-friendly controls such as sliders, scroll menus, and contextual prompts. This ensures that the app can be comfortably used by individuals with varying levels of digital literacy.

#### Clinical Screening Interface

The RiskOCA platform includes a secure, role-specific interface designed for the members of the oral health care team—including licensed dentists and trained VHVs—to facilitate oral cancer screening. To ensure authenticated and traceable access, users must complete a 1-time registration process by providing their full name, national identification number, affiliated health care facility, and, for dentists, their professional license number.

Upon successful login, users are directed to the patient entry screen, where they input the full name and national identification number of the individual undergoing screening. Once patient identification is confirmed, VHVs—under dentist supervision—can record lesion findings by selecting from 9 predefined anatomical sites commonly associated with OPMDs and oral cancer:

1. Upper and lower lips
2. Right and left buccal mucosa
3. Upper and lower gingiva (gums)
4. Retromolar trigone (area behind the last molars)
5. Hard palate
6. Soft palate
7. Dorsal and lateral surfaces of the tongue
8. Ventral (underside) of the tongue
9. Floor of the mouth

Each lesion is then visually categorized into 1 of 5 clinical types:

1. White lesion
2. Red lesion
3. Mixed red and white lesion
4. Ulcerative lesion
5. Mass or lump

**Figure 2.**
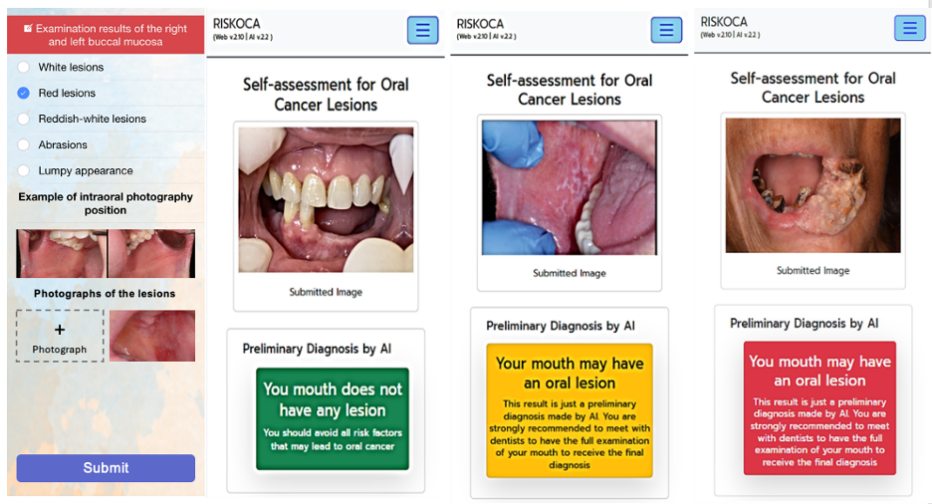
Oral examiner and artificial intelligence (AI) analysis interface.

Photographs of the lesions are captured using a smartphone under natural lighting, with auxiliary light sources used as needed. Images captured were obtained exclusively using mobile devices operated by VHVs. To reduce variability and support consistent image quality, VHVs received structured training on capturing intraoral photographs from 9 predefined anatomical sites, with specific guidance on lighting conditions, image focus, framing, and resolution requirements prior to uploading images to the RiskOCA platform. Despite these measures, differences in clinical context and acquisition conditions between training and field images may influence model performance and are acknowledged as a consideration for generalizability. These images are securely transmitted to the Intercenter Center for Oral Health server, where they undergo AI-based evaluation and expert review to support the referral and care pathway (Figure 2).

#### AI Output and Patient Feedback Interface

Upon image submission, the embedded AI model performs real-time analysis to classify the case into 1 of the 3 risk categories: no visible lesion, suspected OPMD, and suspected OSCC. These preliminary classifications are immediately displayed to the user in the form of a simple, color-coded message and visual overlay indicating lesion location (if applicable). To ensure clinical safety and reduce false positives or negatives, all images—regardless of AI output—are forwarded to participating oral health specialists for manual validation through the dentist interface.

If the AI detects a suspected OPMD or OSCC, the app flags the case and initiates a follow-up protocol. Users with flagged findings receive a notification recommending further evaluation. In parallel, system administrators contact the participant directly and facilitate referral to the nearest designated oral cancer center for in-person examination and, if necessary, biopsy confirmation.

To support public awareness and encourage proactive behavior, the platform also includes embedded educational content, including short videos and visual guides. These materials explain the etiology of oral cancer, common early warning signs (eg, white or red patches, ulcers, and unexplained lumps), and evidence-based self-care recommendations. This dual function—automated risk assessment and patient education—aims to increase health literacy while expediting the path to diagnosis and treatment in high-risk individuals.

#### Expert Review and Diagnostic Confirmation

All images analyzed by the AI model—regardless of classification (normal, OPMD, or OSCC)—were subjected to independent expert review. Two board-certified oral medicine specialists, each with more than 5 years of clinical experience in the diagnosis and management of OPMD and oral cancer, participated in the expert review process.

The specialists independently reviewed all submitted images through a secure clinician portal without access to AI-generated classifications or outputs. Reviews were based on standard clinical visual assessment of intraoral photographs. Cases identified by either the AI model or specialists as suspicious for OPMD or OSCC were referred for further clinical evaluation and biopsy confirmation when indicated.

Among the 1242 adult community members screened, the AI model flagged 8 cases as suspected OPMD and 8 cases as suspected OSCC. Independent specialist review identified 10 cases of OPMD and 6 cases of OSCC. Biopsy-confirmed diagnoses demonstrated that 0.81% (n=10) of the participants had OPMD and 0.48% (n=6) had OSCC with clinically significant lesions.

There were no instances in which normal cases were misclassified as normal by either the AI model or the specialists, and no OSCC cases were missed by either approach. Two cases initially classified by the AI model as OSCC were subsequently identified by specialists and biopsy-confirmed as OPMD, representing the primary source of discordance. No cases classified as normal by the AI model or specialists were later found to harbor a clinically significant disease.

For cases deemed suspicious based on the AI model or specialists, the RiskOCA administrative team contacted the participants and coordinated referral to the nearest designated oral cancer center. There, patients underwent comprehensive clinical evaluation, including biopsy and histopathological analysis, which served as the diagnostic gold standard. Histopathological confirmation allowed for definitive diagnosis, and in several cases, the final outcomes extended beyond the initial screening categories of OPMD and OSCC—highlighting the complexity and spectrum of oral mucosal pathology encountered in community-based screenings. This multitiered workflow—integrating AI triage, expert review, and histological confirmation—ensures clinical accuracy while maintaining accessibility for early detection in at-risk populations.

### User Evaluation

#### Community Participants for Screening

A total of 1242 adult community members residing in the Phu Kamyao district, Phayao province, were recruited to undergo oral cancer screening using the RiskOCA app. This rural district was selected due to its limited access to oral health care services and the high prevalence of behavioral risk factors associated with oral cancer. Participants were eligible if they were aged 40 years or older, had permanent residency in the study area, were capable of providing informed consent, and were able to use a smartphone independently or with assistance from a trained volunteer.

Prior to the screening activities, all VHVs participated in a structured training session designed to ensure safe and consistent use of the RiskOCA app. The training consisted of a 1.5-hour session that included (1) a lecture on basic oral cancer knowledge, common clinical presentations, and associated risk factors; (2) an introduction to the RiskOCA app interface and workflow for risk assessment; and (3) hands-on practice using a mouth simulation model. During the hands-on component, VHVs were trained—under dentist supervision—to capture standardized intraoral photographs and to record lesion-related findings within the RiskOCA app. Following the training, VHVs facilitated participant recruitment, introduced the RiskOCA app, and supported community members during image capture and questionnaire completion as needed.

#### Village Health Volunteers for Usability Evaluation

A total of 250 VHVs participated in the usability evaluation of the RiskOCA app. These VHVs were the frontline users responsible for guiding community members through the self-screening process, and they played a key role in oral cancer risk surveillance in the region.

All VHVs were recruited from the same rural district and had prior experience with basic health education and mobile technology. As part of the study, they received hands-on training in using the app, including functionalities related to user registration, image capture, data entry, and interpretation of AI-generated outputs.

Following their use of the app in real-world screening activities, VHVs completed a structured usability questionnaire designed to assess the app’s interface, functionality, alert system, ease of use, and perceived value for oral cancer surveillance. Their feedback was used to evaluate the app’s practicality, acceptability, and potential for broader community-based deployment.

#### Usability Evaluation

To evaluate the usability and user satisfaction of the RiskOCA app, a structured questionnaire was administered to all participating village health volunteers following their use of the platform. The questionnaire assessed key domains, including interface design, functional performance, automated alert features, ease of use, perceived impact on community surveillance, and overall satisfaction.

User satisfaction and usability were assessed using a 25-item questionnaire adapted from the Mobile App Usability Questionnaire (MAUQ) [16]. The MAUQ aligns with established usability principles, including effectiveness, efficiency, and satisfaction, as outlined in ISO 9241‐210 human-centered design standards. The original MAUQ framework was supplemented with context-specific items addressing oral cancer risk assessment and older adult surveillance to reflect the intended use of the RiskOCA platform in community-based screening. The content validity of the adapted instrument was evaluated by a panel of 6 experts, including public health specialists, oral cancer clinicians, and mHealth or informatics experts. Item-level content validity index values ranged from 0.87 to 0.92, and the scale-level content validity index was 0.91. Minor wording revisions were made following expert review to enhance clarity and contextual appropriateness. A pilot test involving 25 community participants was conducted to assess clarity, feasibility, and reliability. The average time required to complete the questionnaire was approximately 5 minutes, and no major difficulties were reported. Internal consistency reliability was evaluated using Cronbach α, with an overall coefficient of 0.89 and domain-specific values ranging from 0.87 to 0.92, indicating good-to-excellent reliability across all domains.

The instrument was organized into six domains based on thematic relevance:

1. General usage: This domain evaluated users’ perceptions of the screen layout and the logical sequencing of on-screen functions during navigation.
2. Functionality: This domain covered the ability to enter and retrieve data during and after examinations, collect risk factor information, raise user awareness regarding oral cancer, and summarize screening outcomes across all oral anatomical sites.
3. Automated alert system: This domain assessed the system’s ability to generate timely prompts, such as identifying missing or incomplete records, assisting with national identification number verification, and alerting users when data were omitted.
4. Ease of use: Items in this domain focused on minimizing data entry burden, supporting note-taking features for enhanced documentation, and evaluating the system’s technical responsiveness (eg, loading speed and screen transitions).
5. Impact on older adult surveillance: This domain explored the app’s utility in identifying risk factors, reducing the cost of screening and care, and improving data reliability compared to traditional paper–based methods.
6. Overall satisfaction: This final domain gauged users’ general impressions of the app, its usefulness in promoting older adult participation, its effectiveness compared to conventional methods, and their willingness to recommend or reuse the app in future screening activities.

Each item was rated on a 5-point Likert scale, with response options ranging from 1=very dissatisfied, 2=dissatisfied, 3=moderately satisfied, 4=satisfied, to 5=very satisfied. This scale enabled the quantifiable measurement of subjective perceptions regarding the app’s effectiveness, usability, and practical relevance in a community screening context. This comprehensive Likert-scale–based evaluation enabled the robust analysis of the system’s usability across multiple dimensions. The approach also accounted for users with varying levels of digital literacy, including those less experienced with smartphone technology. Mean scores were calculated for each domain as well as for the full scale. Descriptive statistics were used to analyze participant responses across all 25 items. In addition, subgroup analyses were conducted to explore differences in satisfaction scores by age group and self-rated smartphone usage ability. Mean scores across subgroups were compared using one-way ANOVA. Statistical significance was set at *P*<.05. All analyses were conducted using SPSS (version 22.0; SPSS Inc).

## Results

### General Information of App Users

This satisfaction assessment was conducted as part of the project titled “Development of a Mobile Application for Self-Surveillance of Potentially Malignant and Oral Cancer Lesions Among the Elderly and At-Risk Groups.” The evaluation took place at the Phu Kamyao district, Phayao province, Thailand. According to Table 2, all 250 training participants were village health volunteers. The majority (50.8%) of them were aged between 30 and 39 years and had a self-rated ability and experience in using smartphones at a “fair” level (49.6%).

**Table 2.** General information of app users (n=250).

General information	Users, n (%)
Age (y)	
20‐29	25 (10)
30‐39	127 (50.8)
40‐49	54 (21.6)
50‐59	32 (12.8)
≥60	12 (4.8)
Total	250 (100)
Smartphone usage ability and experience	
Beginner	95 (38.0)
Fair	124 (49.6)
Moderate	24 (9.6)
Good	7 (2.8)
Expert	0 (0)
Total	250 (100)

### User Satisfaction Results

The analysis of the mean satisfaction scores across all 6 domains revealed consistently positive user perceptions of the app. The impact on older adult surveillance domain received the highest average score (mean 4.30, SD 0.75), suggesting that users strongly agreed that the app enhanced their ability to identify risk factors, improved data reliability compared to paper-based methods, and contributed to reducing the cost of screening and care. The overall satisfaction domain also achieved a high rating (mean 4.21, SD 0.68), indicating strong endorsement of the app’s usefulness, ease of use, and potential for broader implementation and recommendation (Table 3).

**Table 3.** Percentage of user satisfaction levels toward the oral cancer screening app among older adult participants (n=250).

Section and item		Very dissatisfied, n (%)	Dissatisfied, n (%)	Moderately satisfied, n (%)	Satisfied, n (%)	Very satisfied, n (%)	Average score (out of 5)
General usage							
1. Screen layout		0 (0)	0 (0)	34 (13.6)	153 (61.2)	63 (25.2)	4.12
2. Order of functions displayed		0 (0)	0 (0)	23 (9.2)	182 (72.8)	45 (18.0)	4.09
Subtotal (1-2)		0 (0)	0 (0)	57 (11.4)	335 (67.0)	108 (21.6)	4.10
Functionality							
3. Data entry during screening		0 (0)	0 (0)	56 (22.4)	98 (39.2)	96 (38.4)	4.16
4. Retrieval of past records		0 (0)	0 (0)	55 (22.0)	121 (48.4)	74 (29.6)	4.08
5. Collecting risk factor data		0 (0)	0 (0)	34 (13.6)	108 (43.2)	108 (43.2)	4.30
6. Raising awareness for oral cancer prevention		0 (0)	0 (0)	63 (25.2)	88 (35.2)	99 (39.6)	4.14
Subtotal (3-6)		0 (0)	0 (0)	208 (20.8)	415 (41.5)	377 (37.7)	4.17
Automated notification system							
7. Alerts for data entry issues		0 (0.0)	0 (0)	63 (25.2)	112 (44.8)	75 (30)	4.05
8. Identifying ID and detecting data changes		0 (0)	0 (0)	45 (18.0)	131 (52.4)	74 (29.6)	4.12
9. Alert for incomplete entries		0 (0)	0 (0)	64 (25.6)	101 (40.4)	85 (34.0)	4.08
Subtotal (7-9)		0 (0)	0 (0)	172 (22.93)	344 (45.87)	234 (31.20)	4.08
Ease of use							
10. Minimal data entry required		0 (0)	0 (0)	51 (20.4)	125 (50)	74 (29.6)	4.09
11. Additional notes entry		0 (0)	0 (0)	49 (19.6)	132 (52.8)	69 (27.6)	4.08
12. App responsiveness		0 (0)	0 (0)	45 (18.0)	126 (50.4)	79 (31.6)	4.14
Subtotal (10-12)		0 (0)	0 (0)	145 (19.33)	383 (51.07)	222 (29.6)	4.10
Impact on older adult surveillance							
13. Risk factor screening support		0 (0)	0 (0)	12 (4.8)	110 (44.0)	128 (51.2)	4.46
14. Reducing screening and care costs		0 (0)	0 (0)	70 (28.0)	67 (26.8)	113 (45.2)	4.17
15. Reliability compared to paper-based recording		0 (0)	0 (0)	52 (20.8)	78 (31.2)	120 (48.0)	4.27
Subtotal (13-15)		0 (0)	0 (0)	134 (17.87)	255 (34.0)	361 (48.13)	4.30
Overall satisfaction							
16. Helps assess oral cancer risk		0 (0)	0 (0)	20 (8.0)	145 (58.0)	85 (34.0)	4.26
17. Promotes elderly participation		0 (0)	0 (0)	36 (14.4)	58 (23.2)	156 (62.4)	4.48
18. Improves quality over paper methods		0 (0)	0 (0)	45 (18.0)	126 (50.4)	79 (31.6)	4.14
19. Easy to use		0 (0)	0 (0)	31 (12.4)	119 (47.6)	100 (40)	4.28
20. Willingness to reuse the app		0 (0)	0 (0)	29 (11.6)	78 (31.2)	143 (57.2)	4.46
21. Recommend to 1‐5 colleagues		0 (0)	0 (0)	99 (39.6)	118 (47.2)	33 (13.2)	3.74
22. Recommend to 6‐30 colleagues		0 (0)	0 (0)	38 (15.2)	123 (49.2)	89 (35.6)	4.20
23. Recommend to more than 30 colleagues		0 (0)	0 (0)	9 (3.6)	167 (66.8)	74 (29.6)	4.26
24. Recommend to entire team		0 (0)	0 (0)	30 (12.0)	156 (62.4)	64 (25.6)	4.14
25. Overall satisfaction with the app		0 (0)	0 (0)	25 (10)	155 (62.0)	70 (28.0)	4.18
Subtotal (16-25)		0 (0)	0 (0)	362 (14.48)	1245 (49.8)	893 (35.72)	4.21
Overall mean score							4.17

**Figure 3.**
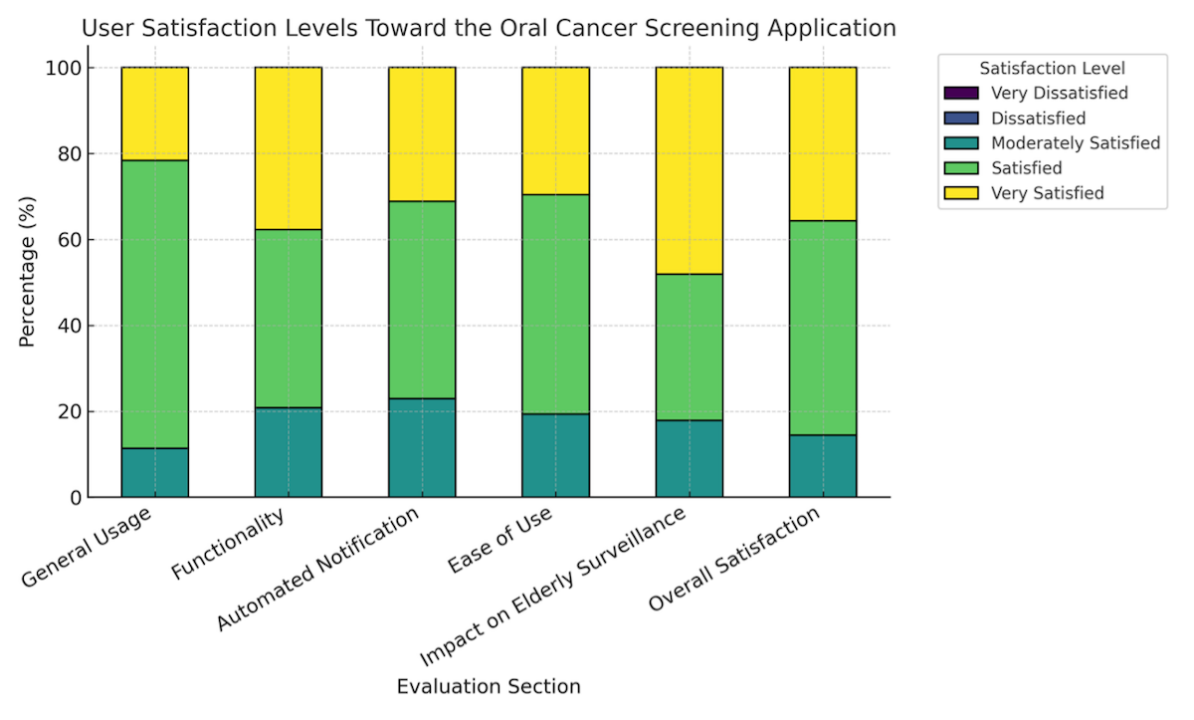
User satisfaction levels toward the oral cancer screening app across 6 evaluation domains (n=250).

The stacked bar chart (Figure 3) illustrates the percentage of older participants reporting different levels of satisfaction with the oral cancer screening app. Responses were collected using a 5-point Likert scale (very dissatisfied to very satisfied) across 6 key domains: general usage, functionality, automated notification, ease of use, impact on older adult surveillance, and overall satisfaction. The majority of users reported being “satisfied” or “very satisfied” in all domains, with the highest satisfaction observed in the “impact on elderly surveillance” domain. No participants reported being dissatisfied or very dissatisfied.

The domains of functionality (mean 4.17, SD 0.75), ease of use (mean 4.10, SD 0.69), and general usage (mean 4.10, SD 0.57) were all rated in the “very good” range, reflecting high user satisfaction with the system’s operational features, visual structure, and user interface design. The automated alert system domain scored slightly lower (mean 4.08, SD 0.73) but still within the “very good” threshold, suggesting that users appreciated the built-in prompts and notifications but may have encountered occasional challenges with error messages or input validation mechanisms.

Overall, the combined mean score across all domains was 4.17 (SD 0.68), indicating a high level of satisfaction among users. These findings affirm that the app was not only functionally effective but also well received by users, including those with limited digital proficiency.

### Subgroup Analysis of User Satisfaction

Subgroup analyses were conducted to examine differences in overall satisfaction scores by age group and self-rated smartphone experience (Table 4). No statistically significant differences in overall satisfaction were observed across age groups or across levels of smartphone experience. These findings indicate that user satisfaction with the RiskOCA app was consistent regardless of age or prior smartphone proficiency. Importantly, mean satisfaction scores across all subgroups remained within the “very good” range, suggesting that the app was well accepted even among VHVs with limited smartphone experience. This consistency supports the usability of the app across a heterogeneous community health workforce.

**Table 4.** Mean overall satisfaction scores by age group and smartphone experience among village health volunteers (n=250)^a^.

Subgroup	Number, n	Mean (SD)	*P* value
Age group (y)			.27
20‐29	25	4.19 (0.71)	
30‐39	127	4.21 (0.85)	
40‐49	54	4.20 (0.75)	
50‐59	32	4.11 (0.62)	
≥60	12	4.15 (0.53)	
Smartphone experience			.31
Beginner	95	4.14 (0.70)	
Fair	124	4.16 (0.63)	
Moderate or good	31	4.21 (0.59)	

a *P* values were calculated using 1-way ANOVA.

### Implementation Challenges and Practical Considerations

Although overall usability was high, several implementation challenges were observed during field deployment. Among the 250 VHVs participating in the usability evaluation, all were able to complete the core screening workflow, including participant data entry, intraoral image capture, and image submission. Based on field observations, the complete screening process was typically completed within approximately 15 minutes per participant, including cases where assistance was required.

No errors in data entry or image submission were observed during the evaluation period. However, a subset of VHVs required assistance when using the RiskOCA app. The most commonly observed challenges included difficulty in capturing adequately focused intraoral images (n=25), inconsistent lighting conditions during image acquisition (n=12), and unfamiliarity with smartphone interface navigation (n=9). In such instances, real-time assistance was provided by supervising dental staff or trained team members, particularly during the initial use of the app.

Minor technical issues, including temporary internet connectivity interruptions and delayed image uploads, were occasionally encountered in areas with unstable network coverage. These issues were typically resolved through repeated upload attempts or by completing submissions once connectivity was restored. No critical system failures, data losses, or breaches of data integrity were observed during the study period.

## Discussion

This study presents a comprehensive evaluation of the technical development and usability of RiskOCA, a smartphone-based oral cancer screening app deployed among VHVs in a rural Thai setting. The findings demonstrate that the app is both feasible and well-accepted, with consistently high satisfaction scores across domains, such as functionality, ease of use, and overall system performance. Importantly, the platform was designed not only as a digital tool but as part of a broader community–based strategy to strengthen the early detection of oral potentially malignant disorders and oral cancer in resource-limited settings.

User acceptance is a critical determinant of success in the development and deployment of mHealth apps [17]. Even technically robust systems may fail to achieve impact if end users find them difficult to use, culturally inappropriate, or poorly integrated into their daily workflows. Previous studies on digital health tools have highlighted that usability, perceived usefulness, and trust strongly influence adoption, particularly in community-based and resource-limited settings [18,19]. In the context of oral cancer screening, where nonspecialist users, such as VHVs, play a central role, ensuring high levels of satisfaction and ease of use is essential for long-term sustainability. The positive feedback from VHVs in this study, therefore, provides an encouraging indication that RiskOCA is acceptable to frontline health workers and has the potential to be successfully scaled in real-world public health systems.

The involvement of VHVs as primary users underscores the value of leveraging task-shifting models in public health. Thailand has long integrated VHVs into its universal health coverage framework, and their role in RiskOCA parallels the use of community health workers in other global initiatives, such as mobile-based cervical cancer screening in sub-Saharan Africa [20] or breast cancer awareness apps in South Asia [21]. Similar to these initiatives, RiskOCA illustrates how mHealth interventions can expand screening coverage, raise the awareness of modifiable risk factors, and create a direct digital pathway between at-risk populations and oral health specialists.

From an implementation perspective, RiskOCA was designed as a modular mHealth platform to support scalability and future functional extensions beyond oral cancer triage. Key design considerations include a flexible system architecture, standardized data structures, and role-based user interfaces that can accommodate additional AI models, clinical workflows, and screening use cases. This design approach enables the potential integration of AI-assisted screening for other oral and maxillofacial conditions, such as oral potentially malignant disorders surveillance, periodontal risk assessment, or referral prioritization for other noncommunicable oral diseases. Anticipating such extensions also underscores the importance of governance frameworks, clinician oversight, and continuous model validation to ensure safety, equity, and clinical relevance as new AI functionalities are introduced within community-based mHealth systems.

A key strength of RiskOCA lies in its potential for scalability and sustainability. The modular architecture allows adaptation across regions, while its integration into existing VHV workflows ensures cost-effectiveness and cultural alignment. Moreover, the dual-layer verification—AI-assisted triage supplemented by universal expert review—enhances diagnostic reliability and reduces the likelihood of false negatives, thereby supporting more timely referral and treatment. By embedding digital innovation into community-driven workflows, RiskOCA has the capacity to reduce diagnostic delays and narrow inequities in specialist access for rural populations.

Despite these strengths, several limitations should be acknowledged. First, only participants flagged as abnormal by the AI system and/or specialists underwent biopsy confirmation; therefore, not all 1242 participants received a gold-standard diagnostic assessment. As a result, true sensitivity and specificity of RiskOCA in the community setting cannot be fully determined, and undetected false-negative cases may exist. Second, variability in image quality—due to differences in lighting, positioning, and smartphone hardware—may affect AI performance. While the deep learning model demonstrated strong accuracy, the greater interpretability of AI outputs is needed to improve user trust and clinical decision support. The current reliance on stable internet connectivity may limit deployment in areas with poor network coverage. Addressing these issues will be critical for large-scale implementation. Another limitation of this study is that usability evaluation relied primarily on self-reported satisfaction measures, and objective usability metrics—such as task completion time per participant, standardized error rates, and formal usability testing protocols—were not systematically recorded. Future studies should incorporate structured usability testing frameworks to quantitatively assess efficiency, learnability, and error recovery, particularly as the platform is scaled to broader community and clinical settings. In addition, this study did not include a direct comparison between AI-assisted screening and traditional oral cancer screening methods conducted by VHVs. Future research should therefore consider randomized controlled trials in which VHVs are assigned to use either the RiskOCA app or conventional visual oral examination protocols. Such comparative designs would allow the rigorous evaluation of screening effectiveness, including detection rates of suspicious lesions, referral accuracy, diagnostic concordance with dentists, and biopsy-confirmed outcomes. Evidence from these trials would be essential to establish the added clinical value of AI-enabled screening over existing community–based practices.

Although a formal cost-effectiveness analysis was beyond the scope of this usability study, the RiskOCA app has the potential to offer cost advantages compared with traditional oral cancer screening pathways. The development of RiskOCA was funded by the Ministry of Public Health of Thailand, with the intention that the app be provided free of charge to users and community health workers. As a result, no direct costs are incurred by villagers or VHVs for app access or routine screening activities. In contrast, traditional oral cancer screening often requires patients to travel to health care facilities or necessitates outreach visits by dentists and health care teams to community settings, both of which involve transportation costs, personnel time, and logistical coordination. By enabling VHVs to conduct preliminary screening using widely available smartphones, RiskOCA may reduce unnecessary referrals, optimize the use of specialist resources, and lower indirect costs associated with delayed diagnosis. Nonetheless, future studies incorporating randomized controlled designs should include formal economic evaluations to quantify cost-effectiveness, taking into account implementation, maintenance, training, and health care system–level outcomes. In addition, structured questionnaire surveys and mixed method evaluations capturing the perspectives, perceived challenges, and recommendations of both VHVs and dentists would provide valuable implementation insights, supporting contextual adaptation and scale-up of similar mHealth initiatives across diverse health care settings.

This study was conducted in a single rural district in northern Thailand, which may limit the generalizability of the findings to other regions with different demographic characteristics, health care infrastructures, digital literacy levels, or patterns of oral cancer risk. Although the setting reflects a high-need population with limited access to oral health care, further studies are warranted to evaluate the performance, usability, and implementation feasibility of the RiskOCA platform in urban settings, other rural regions, and across different health system contexts. Multisite and cross-regional evaluations will be essential to confirm the scalability and adaptability of this community-based AI-assisted screening approach.

Future development should also focus on larger-scale deployment and longitudinal evaluation to validate RiskOCA’s impact on referral rates, confirmed diagnoses, and patient outcomes. Integration with national eHealth records could support coordinated care and surveillance, while the development of multilingual interfaces and culturally adapted educational modules would enhance accessibility for ethnolinguistic minorities. Together, these efforts will be essential to ensuring RiskOCA’s adaptability, effectiveness, and sustainability in diverse low- and middle-income country contexts.

In summary, this study demonstrates that RiskOCA is technically feasible and acceptable to frontline health volunteers. By bridging community-level screening with specialist expertise, the platform represents a scalable and policy-relevant model for strengthening early oral cancer detection in underserved populations. Its successful deployment highlights the broader potential of AI-integrated mHealth systems to transform community-based cancer prevention and control globally.

## Abbreviations

AI: artificial intelligence
MAUQ: Mobile App Usability Questionnaire
mHealth: mobile health
OPMD: oral potentially malignant disorder
OSCC: oral squamous cell carcinoma
PII: personally identifiable information
RiskOCA: Risk Assessment for Oral Cancer using Artificial Intelligence
VHV: village health volunteer

## References

[1] Sung H, Ferlay J, Siegel RL (2021). Global cancer statistics 2020: GLOBOCAN estimates of incidence and mortality worldwide for 36 cancers in 185 countries. CA Cancer J Clin.

[2] Global Cancer Observatory (GCO): Cancer Today. International Agency for Research on Cancer, World Health Organization.

[3] Warnakulasuriya S (2009). Global epidemiology of oral and oropharyngeal cancer. Oral Oncol.

[4] Warnakulasuriya S (2020). Oral potentially malignant disorders: a comprehensive review on clinical aspects and management. Oral Oncol.

[5] Papachristou Nadal I, Aramrat C, Jiraporncharoen W (2021). Process evaluation protocol of a cluster randomised trial for a scalable solution for delivery of Diabetes Self-Management Education in Thailand (DSME-T). BMJ Open.

[6] Nawsuwan K, Singweratham N, Suriya N, Oupra R (2025). Solving area-based problems through intellectual empowerment: a model for developing village health volunteers’ and public health officials’ competencies in producing academic work. Sci Rep.

[7] Nagao T, Warnakulasuriya S (2020). Screening for oral cancer: future prospects, research and policy development for Asia. Oral Oncol.

[8] Kanmodi KK, Salami AA, Shah K, Zohoori FV, Nnyanzi LA (2024). The types and effectiveness of mobile health applications used in improving oral cancer knowledge: a mixed methods systematic review. Health Sci Rep.

[9] Donkor A, Ayitey JA, Adotey PN (2023). Mobile-based application interventions to enhance cancer control and care in low- and middle-income countries: a systematic review. Int J Public Health.

[10] Warin K, Limprasert W, Suebnukarn S, Jinaporntham S, Jantana P, Vicharueang S (2022). AI-based analysis of oral lesions using novel deep convolutional neural networks for early detection of oral cancer. PLoS ONE.

[11] Acharya A, Bhat S, Sultana N (2022). AI-based analysis of oral lesions using novel deep convolutional neural networks for early detection of oral cancer. Oral Oncol.

[12] Guo J, Li B (2018). The application of medical artificial intelligence technology in rural areas of developing countries. Health Equity.

[13] Wongsapai M, Wudtijureepun K, Tamdee D, Suthachai T, Wuttisarnwattana P, Suebnukarn S (2025). Oral cancer surveillance in remote areas using a smartphone digital platform. Stud Health Technol Inform.

[14] Wuttisarnwattana P, Wongsapai M, Theppitak S (2024). Precise identification of oral cancer lesions using artificial intelligence. Stud Health Technol Inform.

[15] Irani S (2020). New insights into oral cancer-risk factors and prevention: a review of literature. Int J Prev Med.

[16] Zhou L, Bao J, Setiawan IMA, Saptono A, Parmanto B (2019). The mHealth app usability questionnaire (MAUQ): development and validation study. JMIR mHealth uHealth.

[17] Schliemann D, Tan MM, Hoe WMK (2022). mHealth interventions to improve cancer screening and early detection: scoping review of reviews. J Med Internet Res.

[18] Holden RJ, Karsh BT (2010). The technology acceptance model: its past and its future in health care. J Biomed Inform.

[19] Adnan A, Irvine RE, Williams A, Harris M, Antonacci G (2025). Improving acceptability of mHealth apps-the use of the technology acceptance model to assess the acceptability of mHealth apps: systematic review. J Med Internet Res.

[20] Brinkel J, Krämer A, Krumkamp R, May J, Fobil J (2014). Mobile phone-based mHealth approaches for public health surveillance in sub-Saharan Africa: a systematic review. Int J Environ Res Public Health.

[21] Hamid F, Roy T (2025). Unveiling sociocultural barriers to breast cancer awareness among the South Asian population: case study of Bangladesh and West Bengal, India. JMIR Hum Factors.

